# Comparison of clinical characteristics and prognosis between type I and type II endometrial cancer: a single-center retrospective study

**DOI:** 10.1007/s12672-023-00820-1

**Published:** 2023-11-23

**Authors:** Yuanpei Wang, Yi Sun, Fangfang Sun, Pin Han, Rujia Fan, Fang Ren

**Affiliations:** 1 Department of Obstetrics and Gynecology, First Affiliated Hospital of Zhengzhou University, Zhengzhou, Henan China; 2 Department of Obstetrics and Gynecology, Luoyang Maternal and Child Health Care Hospital, Luoyang, Henan China; 3 Department of Obstetrics and Gynecology, Henan Province People’s Hospital, Zhengzhou University, Zhengzhou, Henan China

**Keywords:** Endometrial cancer, Type I, Type II, Clinical characteristics, Prognosis

## Abstract

**Objectives:**

To explore the differences in clinical characteristics, prognosis, and risk factors between type I and type II endometrial cancer (EC).

**Materials and methods:**

We retrospectively collected EC patients diagnosed with type I or type II EC from 2009 to 2021 in the First Affiliated Hospital of Zhengzhou University.

**Results:**

In total, 606 eligible EC patients (396 type I, and 210 type II) were included. Baseline analyses revealed that type II patients were older, had more advanced clinical stage, were more likely to receive chemoradiotherapy, and had higher incidence of myometrial infiltration, cervix involvement, lymph node metastasis and positive ascites cytology. Type II significantly favored poorer overall survival (OS) (HR = 9.10, 95%CI 4.79–17.28, *P* < 0.001) and progression-free survival (PFS) (HR = 6.07, 95%CI 2.75–13.37, *P* < 0.001) compared to type I. For all included EC, univariate and multivariate COX analyses revealed age, myometrial infiltration and pathological type were independent risk factors for OS and PFS. Subgroup analyses identified age, menopause, clinical stage, and lymph node metastasis as independent risk factors for type I regarding OS. While age, myometrial infiltration and chemoradiotherapy were identified as risk and protective factors for type II regrading OS. Age and cervix involvement were identified as independent risk factors for type I regarding PFS. Myometrial infiltration was identified as independent risk factor for type II regarding PFS.

**Conclusion:**

Type II patients shared different clinical characteristics and worse prognosis compared to type I, and their independent risk and protective factors also varied.

**Supplementary Information:**

The online version contains supplementary material available at 10.1007/s12672-023-00820-1.

## Introduction

EC remains one of the most common gynecological malignancies, especially in developed counties [1, 2]. It is estimated that there will be 66,200 new EC cases and 13,030 new deaths in the United States in 2023 [3]. According to Bokhman et al. EC patients can be classified as type I and type II subgroups, accounting for approximately 80% and 10–20% of all EC cases, respectively [4, 5]. Usually, type I refers to grade 1/2 uterine endometrioid carcinomas, while type II includes some non- endometrioid carcinomas, mainly including uterine serous carcinoma (10%), uterine clear cell carcinoma (3%), uterine carcinosarcoma (< 2%), uterine undifferentiated carcinoma (1–2%) [6]. Patients with type I EC are estrogen dependent, obese, younger, while patients with type II are older, estrogen independent, and poorly differentiated (grade 3) [7, 8]. Also, EC patients with different pathological types shared different molecular etiology. For example, somatic mutations in PTEN, PIK3CA, and PIK3R1 lead to the progression of endometrioid endometrial cancer. TP53 mutations and/or p53 drive the early carcinogenesis of uterine serous cancer, and high phosphorylation of TP53BP1-S1763 and CHEK2- S163 regulates cell cycle in uterine serous carcinoma. However, most uterine carcinosarcomas simultaneously share both PTEN and TP53 mutations [6, 9–13].

Molecular typing and histology characteristics are two important factors for grouping prognostic risk  in EC patients [14]. Molecular typing independent of histology improves the accuracy and reproducibility of EC diagnosis, which is of great significance for predicting prognosis, guiding treatment, and genetic screening [15]. EC patients with different molecular types share different immune microenvironments. For example, higher infiltration of CD8 + T cells were verified in EC patients with POLEmut and MMRd subtype [16]. The abundance of some common immune checkpoints (e.g., PD-1, PD-L1) varies among different pathological types, and the positive rate of PD-1/PD-L1 in uterine endometrioid carcinomas, uterine serous carcinoma, and uterine clear cell carcinoma are 40–80%, 10–68%, and 23–69%, respectively, which may affect their response to immunotherapy [17, 18].

Type II exhibits more aggressive biological behaviors, such as a higher risk of lymph nodes and distant metastasis, more advanced stage and poorer differentiation, leading to significantly unfavorable prognosis [8, 19]. Previous studies revealed that 5-year survival rate for type I was approaching 85%. However, the long-term prognosis of type II is far from satisfactory, which results in approximately 40% of all EC-related deaths [20–24]. Previous studies revealed the 5-year OS for uterine serous carcinoma, uterine carcinosarcoma, and uterine clear cell carcinoma was 45.9%, 53.6%, and 63%, respectively, which was far lower than that in type I [25–27]. Currently, differences in clinical characteristics, prognosis, and treatment regimens between type I and type II have not been well elucidated for limited samples were included in previous studies. Therefore, it is great important to identify risk factors for carcinogenesis and prognosis to develop optimal treatments for type II with a larger sample size.

In this study, we retrospectively collected eligible EC patients with type I or type II to compare their differences in demographic characteristics and prognosis. Specific independent prognostic factors were also identified for type I and type II. This study will provide suggestions for endometrial cancer patients with different pathological types and clinical features to make appropriate clinical decisions.

## Material and methods

### Patients cohort

This retrospective study was approved by the ethics committee of First Affiliated Hospital of Zhengzhou University (Approved number: 2023-KY-0350-002). Patients diagnosed with EC between 2009 and 2021 in the department of obstetrics and gynecology of First Affiliated Hospital of Zhengzhou University were retrospectively collected. Type II EC included uterine serous carcinoma, uterine mixed carcinoma, uterine clear cell carcinoma, uterine undifferentiated carcinoma, and uterine carcinosarcoma. While type I EC only contained grade 1/2 uterine endometrioid carcinomas. The inclusion criteria were as follows: (1) EC with unambiguous pathological types mentioned above; (2) EC with accurate OS data; (3) EC without simultaneous diagnosis of other malignant tumors. The pathological diagnosis of all included patients was confirmed by two senior pathologists independently. The data of patients' clinical stage were measured according to the 2009 modified International Federation of Gynecology and Obstetrics (FIGO) system.

The following data of demography (e.g. age, pathological types, grade, body weight and height, treatment programs, status of myometrial infiltration, lymph node metastasis, and cervix involvement, clinical stage, etc.) and follow-up (e.g. status of survival or recurrence, clear time of death or recurrence) were collected to further performed analysis. Myometrial infiltration (≥ 1/2) was regarded as deep infiltration.

### Analysis of clinical data

OS, referring to the time from diagnosis to the last follow-up or death, was regarded as the primary endpoint. While PFS, which referrers to the time from diagnosis to the last follow-up or recurrence, was regarded as the second endpoint. The log-rank test was used to measure the statistical differences of different pathological type on OS and PFS. The univariate and multivariate Cox models were used to identify the independent prognostic factors for EC patients.

### Statistics

All the statistical analyses were performed using the R software (version 4.2.1). The differences in baseline characteristics between type I and type II EC patients were measured using the R package stats (version 4.2.1). The Kaplan–Meier curves were performed using the R package survival (version 3.3.1) and R package survminer. The 5-year survival rate was calculated by the SPSS software (version: 26.0, SPSS, Inc). The univariate and multivariate Cox models were performed using the R package survival (version 3.3.1) and R package rms (version 6.3-0). In our study, *P* < 0.05 was considered statistically significant.

## Results

### Characteristics of included patients

In total, 606 eligible patients diagnosed with EC from 2009 to 2021 were included. Among them, 396 patients were type I (236 grade 1 uterine endometrioid carcinomas, accounting for 59.60%; 160 grade 2 uterine endometrioid carcinomas, accounting for 40.40%), and 210 patients were type II (106 uterine serous carcinomas, accounting for 50.48%; 34 uterine mixed carcinomas, accounting for 16.19%; 34 uterine clear cell carcinomas, accounting for 16.19%; 18 uterine undifferentiated carcinomas, accounting for 8.57%; and 18 uterine carcinosarcomas, accounting for 8.57%). Significant differences were found between type I and type II EC cohorts regarding baseline demographics (Table 1). Compared to type I, type II patients were older (60.367 vs. 54.705 years) and menopausal (90.5% vs. 59.3%), were more likely to receive chemoradiotherapy (37.6% vs. 8.3%), were in a more advanced stage (stage III/IV: 25.7% vs. 7.3%) and poorer differentiation (Grade 3/4: 84.3% vs. 0%), were more susceptible to deep myometrial infiltration (36.2% vs. 17.7%), cervix involvement (14.8% vs. 4.5%), and lymph node metastasis (20.5% vs. 4.8%), and positive ascites cytology (9.5% vs. 2.3%). Overall, Type II patients achieved shorter time of OS (1199.5 vs. 1669.5 days) and PFS (1280 vs. 1677 days), and type II patients significantly favored poorer OS and PFS compared to those with type I (Supplementary Fig. 1A and B). Further analysis revealed that the 5-year survival rates regarding OS in all stage I/II/III/IV EC patients were 93.6%, 88.2%, 75.4%, 32.7%, respectively. The 5-year survival rates regarding PFS in all stage I/II/III/IV EC patients were 95.0%, 84.4%, 87.6%, and 85.7%, respectively. Furthermore, the 5-year survival rate (OS) of type I was significantly higher than that of type II in stage I (96.9% vs. 83.6%, *P* < 0.001) and stage III (91.2% vs. 63.8%, *P* = 0.004) (Table 2). We also compared the prognostic differences within type II EC. As shown in Supplementary Fig. 1C and D, only uterine mixed carcinoma obtained better OS compared to uterine serous carcinoma (HR = 0.11706, 95%CI 0.016–0.866, *P* = 0.0356).

**Table 1:** Baseline characteristics of included EC patients with different pathological types

Characteristics	Type I	Type II	*P*-value
n	396	210	
Age, mean ± sd	54.705 ± 8.7763	60.367 ± 8.5512	**< 0.001**
Menopause, n (%)			**< 0.001**
Yes	235 (59.3%)	190 (90.5%)	
No	150 (37.9%)	19 (9%)	
Unknown	11 (2.8%)	1 (0.5%)	
BMI, median (IQR)	25.462 (23.422, 28.134)	25.036 (22.656, 28.012)	0.236
Grade, n (%)			**< 0.001**
G1	236 (59.6%)	0 (0%)	
G2	160 (40.4%)	33 (15.7%)	
G3	0 (0%)	159 (75.7%)	
G4	0 (0%)	18 (8.6%)	
Surgery, n (%)			**0.003**
No	0 (0%)	6 (2.9%)	
Yes	396 (100%)	204 (97.1%)	
Chemotherapy alone, n (%)			0.411
No	264 (66.7%)	133 (63.3%)	
Yes	132 (33.3%)	77 (36.7%)	
Radiotherapy alone, n (%)			1.000
No	393 (99.2%)	208 (99%)	
Yes	3 (0.8%)	2 (1%)	
Chemoradiotherapy, n (%)			**< 0.001**
No	363 (91.7%)	131 (62.4%)	
Yes	33 (8.3%)	79 (37.6%)	
Without systemic therapy, n (%)			**< 0.001**
No	168 (42.4%)	158 (75.2%)	
Yes	228 (57.6%)	52 (24.8%)	
Stage, n (%)			**< 0.001**
I	361 (91.2%)	138 (65.7%)	
II	6 (1.5%)	11 (5.2%)	
III	27 (6.8%)	42 (20%)	
IV	2 (0.5%)	12 (5.7%)	
Unknown	0 (0%)	7 (3.3%)	
Myometrial infiltration (> 1/2), n (%)			**< 0.001**
No	297 (75%)	118 (56.2%)	
Yes	70 (17.7%)	76 (36.2%)	
Unknown	29 (7.3%)	16 (7.6%)	
Cervix involvement, n (%)			**< 0.001**
No	333 (84.1%)	170 (81%)	
Yes	18 (4.5%)	31 (14.8%)	
Unknown	45 (11.4%)	9 (4.3%)	
Lymph node metastasis, n (%)			**< 0.001**
No	299 (75.5%)	141 (67.1%)	
Yes	19 (4.8%)	43 (20.5%)	
Unknown	78 (19.7%)	26 (12.4%)	
Ascites cytology, n (%)			**< 0.001**
Negative	374 (94.4%)	190 (90.5%)	
Positive	9 (2.3%)	20 (9.5%)	
Unknown	13 (3.3%)	0 (0%)	
OS, n (%)			**< 0.001**
Alive	384 (97%)	166 (79%)	
Dead	12 (3%)	44 (21%)	
OS-time(days), median (IQR)	1669.5 (1392.5, 2134)	1199.5 (794.5, 1795)	**< 0.001**
PFS, n (%)			**< 0.001**
Stable	380 (96%)	155 (73.8%)	
Recurrent	9 (2.3%)	21 (10%)	
Unknown	7 (1.8%)	34 (16.2%)	
PFS-time(days), median (IQR)	1677 (1393, 2134)	1280 (878, 1880.5)	**< 0.001**

*EC* endometrial carcinoma, *BMI* body mass index, *OS* overall survival, *PFS* progression-free survival

**Table 2:** 5-year survival rate of different EC cohorts with different pathological type at different stages

Stage	OS				PFS			
	Overall	Type I	Type II	*P*-value	Overall	Type I	Type II	*P*-value
I	93.6	96.9	83.6	**< 0.001**	95.0	96.9	88.9	**< 0.001**
II	88.2	NA	81.8	0.284	84.4	NA	75.0	0.260
III	75.4	91.2	63.8	**0.004**	87.6	96.2	80.3	0.075
IV	32.7	50	57.1	0.871	85.7	NA	83.3	0.683

*EC* endometrial cancer, *OS* overall Survival, *PFS* progression-free survival, *NA* not available

### Univariate and multivariate analyses of all EC patients

We then performed univariate and multivariate Cox analyses to identify independent prognostic factors for all included patients. For OS, age (HR = 1.067, 95%CI 1.029–1.108, *P* < 0.001), deep myometrial infiltration (HR = 2.967, 95%CI 1.496–5.885, *P* = 0.002), and pathological type (HR = 10.620, 95%CI 4.081–27.635, *P* < 0.001) were independent risk factors for all EC cohort (Table 3). While for PFS, age (HR = 1.081, 95%CI 1.019–1.146, *P* = 0.010), deep myometrial infiltration (HR = 2.976, 95%CI 1.208–7.335, *P* = 0.018), and pathological type (HR = 7.466, 95%CI 2.237–24.922, *P* = 0.001) were independent risk factors for all EC cohort (Table 4).

**Table 3:** Univariate and multivariate Cox regression analysis for OS

Characteristics	No	Univariate analysis		Multivariate analysis	
		Hazard ratio (95% CI)	*P-*value	Hazard ratio (95% CI)	*P-*value
Age	606	1.097 (1.067–1.128)	**< 0.001**	1.067 (1.029–1.108)	**< 0.001**
Menopause	606				
No	169	Reference		Reference	
Yes	425	5.676 (2.052–15.699)	**< 0.001**	1.026 (0.314–3.350)	0.966
Unknown	12	0.000 (0.000–Inf)	0.996	0.000 (0.000–Inf)	0.996
BMI	284	1.061 (0.972–1.158)	0.186		
Surgery	606				
Yes	600	Reference		Reference	
No	6	4.984 (1.209–20.553)	**0.026**	0.162 (0.017–1.538)	0.113
Chemotherapy alone	606				
No	397	Reference			
Yes	209	0.711 (0.398–1.271)	0.250		
Radiotherapy alone	606				
No	601	Reference			
Yes	5	0.000 (0.000–Inf)	0.995		
Chemoradiotherapy	606				
Yes	112	Reference			
No	494	0.645 (0.352–1.181)	0.155		
Without systemic therapy	606				
No	326	Reference			
Yes	280	1.043 (0.616–1.766)	0.875		
Stage	606				
I	499	Reference		Reference	
II	17	2.204 (0.524–9.272)	0.281	1.188 (0.234–6.035)	0.835
III	69	5.050 (2.717–9.384)	**< 0.001**	1.023 (0.247–4.241)	0.975
IV	14	21.986 (9.700–49.832)	**< 0.001**	3.703 (0.930–14.740)	0.063
Unknown	7	13.111 (3.944–43.586)	**< 0.001**	2.100 (0.198–22.223)	0.538
Myometrial infiltration (> 1/2)	606				
No	415	Reference		Reference	
Yes	146	6.090 (3.402–10.904)	**< 0.001**	2.967 (1.496–5.885)	**0.002**
Unknown	45	3.136 (1.155–8.512)	**0.025**	0.785 (0.130–4.740)	0.792
Cervix involvement	606				
No	503	Reference		Reference	
Yes	49	3.890 (2.022–7.484)	**< 0.001**	0.806 (0.353–1.843)	0.610
Unknown	54	2.256 (1.047–4.860)	**0.038**	6.938 (1.738–27.690)	**0.006**
Lymph node metastasis	606				
No	440	Reference		Reference	
Yes	62	8.786 (4.818–16.019)	**< 0.001**	3.429 (0.880–13.370)	0.076
Unknown	104	2.664 (1.342–5.291)	**0.005**	2.951 (1.277–6.819)	**0.011**
Ascites cytology	606				
Negative	564	Reference		Reference	
Positive	29	3.015 (1.358–6.691)	**0.007**	1.040 (0.419–2.581)	0.932
Unknown	13	4.354 (1.337–14.176)	**0.015**	58.806 (12.648–273.408)	**< 0.001**
Pathological type	606				
Type I	396	Reference		Reference	
Type II	210	9.099 (4.791–17.278)	**< 0.001**	10.620 (4.081–27.635)	**< 0.001**

*BMI* body mass index, *OS* overall survival, *CI* confidence interval

**Table 4:** Univariate and multivariate Cox regression analysis for PFS

Characteristics	No	Univariate analysis		Multivariate analysis	
		Hazard ratio (95% CI)	*P-*value	Hazard ratio (95% CI)	*P-*value
Age	564	1.094 (1.053–1.138)	**< 0.001**	1.081 (1.019–1.146)	**0.010**
Menopause	564				
No	164	Reference		Reference	
Yes	389	3.873 (1.172–12.799)	**0.026**	0.611 (0.140–2.676)	0.513
Unknown	11	0.000 (0.000–Inf)	0.997	0.000 (0.000–Inf)	0.999
BMI	262	0.978 (0.871–1.098)	0.704		
Surgery	564				
No	4	Reference			
Yes	560	3303224.2846 (0.000–Inf)	0.997		
Chemotherapy alone	564				
No	371	Reference			
Yes	193	0.575 (0.245–1.346)	0.202		
Radiotherapy alone	564				
No	559	Reference			
Yes	5	0.000 (0.000–Inf)	0.997		
Chemoradiotherapy	564				
Yes	105	Reference		Reference	
No	459	0.255 (0.122–0.530)	**< 0.001**	0.726 (0.259–2.035)	0.542
Without systemic therapy	564				
No	303	Reference		Reference	
Yes	261	0.522 (0.237–1.147)	0.105	1.689 (0.530–5.380)	0.375
Stage	564				
I	480	Reference		Reference	
II	16	3.168 (0.738–13.602)	0.121	0.915 (0.126–6.630)	0.930
III	57	3.423 (1.438–8.146)	**0.005**	0.000 (0.000–Inf)	0.998
IV	7	4.959 (0.657–37.413)	0.120	0.000 (0.000–Inf)	0.998
Unknown	4	0.000 (0.000–Inf)	0.997	0.088 (0.000–Inf)	1.000
Myometrial infiltration (> 1/2)	564				
No	402	Reference		Reference	
Yes	122	5.601 (2.644–11.863)	**< 0.001**	2.976 (1.208–7.335)	**0.018**
Unknown	40	0.000 (0.000–Inf)	0.997	0.000 (0.000–Inf)	0.999
Cervix involvement	564				
No	474	Reference		Reference	
Yes	42	8.114 (3.680–17.891)	**< 0.001**	3.138 (0.785–12.543)	0.106
Unknown	48	1.873 (0.545–6.432)	0.319	5.997 (1.112–32.351)	**0.037**
Lymph node metastasis	564				
No	425	Reference		Reference	
Yes	46	4.833 (2.098–11.134)	**< 0.001**	7.68E + 08 (0.000–Inf)	0.998
Unknown	93	0.781 (0.230–2.652)	0.692	1.094 (0.281–4.260)	0.897
Ascites cytology	**564**				
Positive	22	Reference		Reference	
Negative	530	15448863.8425 (0.000–Inf)	0.996	3.98E + 08 (0.000–Inf)	0.999
Unknown	12	70761583.0346 (0.000–Inf)	0.996	3.81E + 09.9671 (0.000–Inf)	0.999
Pathological type	564				
Type I	389	Reference		Reference	
Type II	175	6.068 (2.754–13.370)	**< 0.001**	7.466 (2.237–24.922)	**0.001**

*BMI* body mass index, *PFS* progression free survival, *CI* confidence interval

### Identification of independent prognostic factors for type I and type II patients

As there were great differences in epidemiology and biological behavior between type I and type II patients, we aimed to further identify specific risk/protective factors for EC with different pathological types. In type I EC, age (HR = 1.251, 95%CI 1.155–1.355, *P* < 0.001), menopause (HR = 1.39E + 04, 95%CI 872.76–2.23E + 05, *P* < 0.001), late stage (stage III/IV) (*P* < 0.001), and lymph node metastasis (HR = 4.86E + 19, 95%CI 1.18E + 19–1.99E + 20, *P* < 0.001) were independent risk factors for OS (Table 5). While age (HR = 1.154, 95%CI 1.060–1.256, *P* < 0.001), cervix involvement (HR = 32.147, 95%CI 6.163–167.688, *P* < 0.001) were the independent risk factors for PFS, and chemotherapy (HR = 0.119, 95%CI 0.014–0.978, *P* = 0.048) was the independent protective factor for PFS (Supplementary Table 1).

**Table 5:** Univariate and multivariate Cox regression analysis for OS in type I EC

Characteristics	No	Univariate analysis		Multivariate analysis	
		Hazard ratio (95% CI)	*P*	Hazard ratio (95% CI)	*P*
Age	396	1.131 (1.067–1.200)	**< 0.001**	1.251 (1.155–1.355)	**< 0.001**
Menopause	396		**0.036**		
No	150	Reference		Reference	
Yes	235	7.055 (0.911–54.646)	0.061	1.39E + 04 (872.755–2.23E + 05)	**< 0.001**
Unknown	11	0.000 (0.000–Inf)	0.998	0.219 (0.000–Inf)	0.997
BMI	125	1.208 (0.961–1.517)	0.105		
Chemotherapy alone	396				
Yes	132	Reference			
No	264	2.890 (0.631–13.226)	0.172		
Chemoradiotherapy alone	396		0.436		
No	363	Reference			
Yes	33	2.147 (0.470–9.802)	0.324		
Without systemic therapy					
No	168	Reference			
Yes	228	1.708 (0.512–5.695)	0.384		
Stage	396		0.059		
I	361	Reference		Reference	
II	6	0.000 (0.000–Inf)	0.997	0.000 (0.000–Inf)	0.985
III	27	2.741 (0.598–12.560)	0.194	0.000 (0.000–0.000)	**< 0.001**
IV	2	57.017 (6.764–480.644)	**< 0.001**	0.000 (0.000–0.000)	**< 0.001**
Myometrial infiltration (> = 1/2)	396				
No	297	Reference		Reference	
Yes	70	6.805 (1.988–23.290)	**0.002**	2.471 (0.735–8.305)	0.144
Unknown	29	2.773 (0.309–24.846)	0.362	0.260 (0.032–2.105)	0.207
Cervix involvement	396				
No	333	Reference		Reference	
Yes	18	5.647 (1.139–28.010)	**0.034**	0.212 (0.046–0.987)	0.048
Unknown	45	5.305 (1.494–18.842)	**0.010**	30.396 (8.747–105.618)	< 0.001
Lymph node metastasis	396		**0.015**		
No	299	Reference		Reference	
Yes	19	9.500 (2.269–39.775)	**0.002**	4.86E + 19 (1.18E + 19–1.99E + 20)	**< 0.001**
Unknown	78	3.218 (0.864—11.987)	0.082	12.944 (3.771–44.425)	**< 0.001**
Ascites cytology	396				
No	374				
Yes	9	4.477 (0.557–35.958)	0.158	0.000 (0.000–0.001)	**< 0.001**
Unknown	13	26.634 (5.888–120.486)	< 0.001	607.104 (130.796–2817.952)	**< 0.001**

*BMI* body mass index, *OS* overall survival, *CI* confidence interval

In type II EC, chemoradiotherapy (HR = 0.472, 95%CI 0.230–0.969, *P* = 0.041) was the protective factor for OS, while age (HR = 1.044, 95%CI 1.004–1.085, *P* = 0.029) and deep myometrial infiltration (HR = 2.965, 95%CI 1.402–6.270, *P* = 0.004) were the independent risk factors (Table 6). For PFS, deep myometrial infiltration (HR = 3.992, 95%CI 1.103–8.115, *P* = 0.031) was the only independent risk factor (Supplementary Table 2).

**Table 6:** Univariate and multivariate Cox regression analysis for OS in type II EC

Characteristics	No	Univariate analysis		Multivariate analysis	
		Hazard ratio (95% CI)	*P-*value	Hazard ratio (95% CI)	*P-*value
Age	210	1.054 (1.018–1.091)	**0.003**	1.044 (1.004–1.085)	**0.029**
Menopause	210				
No	19	Reference			
Yes	190	1.621 (0.498–5.276)	0.422		
Unknown	1	0.000 (0.000–Inf)	0.996		
BMI	159	1.081 (0.972–1.202)	0.150		
Surgery	210				
No	6	Reference			
Yes	204	0.555 (0.134–2.306)	0.418		
Chemotherapy alone	210				
No	133	Reference			
Yes	77	0.868 (0.460–1.639)	0.663		
Radiotherapy alone	210				
No	208	Reference			
Yes	2	0.000 (0.000–Inf)	0.997		
Chemoradiotherapy	210				
No	131	Reference		Reference	
Yes	79	0.546 (0.281–1.061)	0.074	0.472 (0.230–0.969)	**0.041**
Stage	210				
I	138	Reference		Reference	
II	11	1.380 (0.320–5.950)	0.666	1.267 (0.218–7.370)	0.792
III	42	3.205 (1.584–6.482)	**0.001**	1.067 (0.249–4.577)	0.930
IV	12	8.164 (3.302–20.186)	**< 0.001**	3.214 (0.784–13.177)	0.105
Unknown	7	4.559 (1.333–15.599)	**0.016**	0.262 (0.023–2.953)	0.279
Myometrial infiltration (> 1/2)	210				
No	118	Reference		Reference	
Yes	76	3.683 (1.900–7.140)	**< 0.001**	2.965 (1.402–6.270)	**0.004**
Unknown	16	2.899 (0.944–8.901)	0.063	0.000 (0.000–Inf)	0.996
Cervix involvement	210				
No	170	Reference		Reference	
Yes	31	2.093 (1.022–4.289)	**0.044**	0.879 (0.358–2.159)	0.778
Unknown	9	3.631 (1.271–10.373)	**0.016**	1.41E + 08 (0.000–Inf)	0.996
Lymph node metastasis	210				
No	141	Reference		Reference	
Yes	43	4.727 (2.420–9.232)	**< 0.001**	2.789 (0.69–11.271)	0.150
Unknown	26	4.026 (1.788–9.063)	**< 0.001**	2.148 (0.716–6.444)	0.173
Ascites cytology	210				
No	190	Reference			
Yes	20	1.423 (0.600–3.372)	0.423		

*BMI* body mass index, *OS* overall survival, *CI* confidence interval

### Subgroup analysis for stage I type II EC patients

Whether patients in early stage with type II EC could benefit from postoperative chemotherapy/radiotherapy or not remains unclear. To measure the impact of postoperative adjuvant therapy on stage I type II EC patients, we further performed univariate and multivariate Cox analyses on these patients. As shown in Supplementary Table 3, only age (HR = 1.089, 95%CI 1.008–1.177, *P* = 0.030) and BMI (HR = 1.388, 95%CI 1.083–1.780, *P* = 0.010) were the independent risk factors for OS in stage I type II EC. However, chemotherapy alone (HR = 0.512, 95%CI 0.168–1.561, *P* = 0.239) or chemoradiotherapy (HR = 0.588, 95%CI 0.208–1.659, *P* = 0.316) did not significantly affect OS of the patients in stage I type II EC. Chemotherapy alone (HR = 0.350, 95%CI 0.075–1.624, *P* = 0.180) or chemoradiotherapy (HR = 0.484, 95%CI 0.147–1.590, *P* = 0.232) also did not significantly affect PFS of the patients in stage I type II EC (Supplementary Table 4).

## Discussion

Our single center retrospective study collected 606 EC to compare the baseline characteristics between type I and type II, and further identify their specific prognostic factors. Compared to type II EC, we found that EC patients with type I were younger and premenopausal, had earlier clinical stage (stage I or II), were less likely to receive chemoradiotherapy, better differentiation, and had higher incidence of lesions confined to uterus, which was consistent with some previous studies [21, 28]. For the entire EC population, age, deep myometrial infiltration, and pathological type were identified as the risk factors for OS and PFS. All these identified prognostic risk factors were consistent with previous studies [29]. It was worth noting that we found clinical stage was not an independent risk factor for prognosis, which was not consistent with previous studies [30, 31]. In our study, the prognosis of type II was far worse than that of type I, even if type II patients were diagnosed with early stage (I/II), which had a 70.9% percentage of the type II. The overall mortality or recurrence rates of early type II patients were 13.42% (20/149) and 9.40% (14/149) during the follow-up period, respectively, which could explain why clinical stage was not a significant factor for prognosis. We also found that the prognostic risk factors also varied greatly between these two different EC subtypes. In type I cohort, age, menopause status, clinical stages, and lymph nodes metastasis were independent risk factors for OS, while age and cervix involvement remained the independent risk factors for PFS. In type II cohort, chemoradiotherapy and deep myometrial infiltration were independent protective and risk factors for OS, respectively. While only deep myometrial infiltration remained the independent risk factor for PFS. In clinical practice, different prognostic risk factors of type I and type II could provide guidance on patient prognostic evaluation and treatment plan selection.

According to the National Comprehensive Cancer Network (NCCN) guidelines, whether EC patients receive post-adjuvant chemotherapy or radiotherapy mainly depends on risk factors, such as age ≥ 60 years old, deep myometrial infiltration, and/or lymphatic vessel space infiltration (LVSI), etc. In our study, the proportion of type II patients (77/210) receiving chemotherapy alone was similar with that of type I patients (132/396). However, the proportion of type II patients (79/210) receiving chemoradiotherapy was significantly higher than that of type I patients (33/396). We found that patients with type I EC could benefit from chemotherapy alone regrading PFS, and patients with type II EC could benefit from chemoradiotherapy regrading OS, which was consistent with some previous studies [32–34]. The proportion of patients receiving radiotherapy alone was extremely low either in type I (3/396) or type II EC (2/210) cohort. Therefore, radiotherapy alone was not included in subsequent univariate and multivariate COX analyses in our study. In summary, these findings will provide suggestions for endometrial cancer patients with different pathological types and clinical features to select appropriate post-adjuvant treatments.

Obesity as a high-risk factor for the carcinogenesis and unfavorable prognosis of EC, especially for type I EC, has been confirmed in many previous studies [35]. Likewise, in our study, we found that body mass index (BMI) was an independent risk factor for OS in type I EC. However, further exploration revealed that the data of BMI was missing in 68% (271/396) of type I patients, which should be further addressed in future studies. In contrast, type II EC cohort had relatively complete data of BMI, with a missing rate of 24% (51/210). Unlike type I EC, the impact of obesity in type II remains unclear [36, 37]. Interestingly, we found that BMI was also not an independent risk factor for type II EC, which was consistent with the study by Caroline et al*.* 2016 [38]. Whether obesity will affect the prognosis of patients with type II EC requires further exploration in the future.

Positive ascites cytology suggests that patients may develop extrauterine and abdominal metastatic diseases, and its positive rate may be influenced by the disease state itself, preoperative laparoscopy or hysteroscopy, and surgical modality [39]. Overall, the positive rate of positive ascites cytology is relatively low and shows a gradually decreasing trend [40]. In our study, the positive rates of ascites in patients with type I and type II EC were 2.27% (9/396) and 9.52% (20/210), respectively. Whether it can serve as an independent prognostic risk factor for patients and affect their treatment plan is still uncertain, and ascites cytology was removed from the FIGO 2009 guidelines for this reason [39, 41, 42]. However, despite this, many clinical guidelines still recommend reporting the results of ascites cytology as a pathological outcome [43]. In our study, the cytological status of ascites cytology did not affect the OS of all included EC population, type I and II EC patients. Due to the limited number of EC included and the low positive rate of ascites cytology, its impact on the prognosis of EC patients in different clinical stages or risk groups needs to be further explored by more patients from different clinical centers in the future.

The molecular typing of EC is a new classification method based on immunohistochemistry (IHC) and DNA sequencing to provide guidance on the prognosis and treatment of patients [15]. In 2013, Douglas et al. divided EC patients into the following four groups based on whole genome sequencing: DNA polymerase ϵ (POLE) mutated, microsatellite instability high (MSI-H), copy number low, and copy number high [44]. However, due to the high cost and high technical requirements of whole genome sequencing, *Talhouk* et al. divided EC patients into the following four groups based on IHC and targeted DNA sequencing in 2015: mismatch repair deficient (MMRd), POLE exonuclease domain mutant (POLEmut), p53 wild type/nonspecific molecular profile (NSMP), and p53 abnormal (p53abn) [45]. At present, molecular typing has been adopted by the NCCN guidelines and the Europe's Leading Gynaecological Oncology Congress (ESGO) in 2020 and 2021, respectively [14, 46]. However, due to factors such as technological limitations and economic costs, the molecular typing methods for EC have not yet been perfected and widely popularized in developed countries. The patients in our study were diagnosed from 2009 to 2021, and the data of molecular typing was serious missing. Molecular classification is increasingly important for prognosis and treatment decisions. In future research, we will combine molecular typing and pathological type for further analysis, aiming to provide more appropriate guidance for clinical practice.

In summary, there are some limitations that should be further resolved in the near future. Firstly, limited samples were included in this single center study, which could result in some no statistical differences and selection bias. More samples from other centers should be included to verify our findings in the near future. Secondly, some pivotal data (e.g., status of lymphovascular space invasion, BMI etc.) were missing in most included patients. Thirdly, we could not compare the differences of molecular classification between type I and type II due to the lack of corresponding data, which was a key factor affecting patients’ drug response and prognosis. Last but not least, no obvious prognostic differences were found within type II EC. Each pathological type should include more samples to compare their prognostic differences in the future.

In summary, the baseline characteristics and prognostic factors of patients with type I were remarkably different from those with type II, and patients with type II obtained unfavorable prognosis compared to those with type I.

### Supplementary Information

Additional file1 (TIF 861 KB): Figure 1. Prognostic analysis of EC patients with different pathological type from our single center. (A) Prognostic difference between type I and type II EC patients regarding overall survival. (B) Prognostic difference between type I and type II EC patients regarding progression-free survival. (C) Prognostic difference between uterine serous carcinoma and other pathological types regarding overall survival. (D) Prognostic difference between uterine serous carcinoma and other pathological types regarding progression free survival

Additional file2 (DOCX 23 KB)

Additional file3 (DOCX 23 KB)

Additional file4 (DOCX 21 KB)

Additional file5 (DOCX 21 KB)

## Data Availability

All data included in this study are available upon request by contact with the corresponding author.

## Abbreviations

EC: Endometrial carcinoma
OS: Overall survival
PFS: Progression-free survival
FIGO: International Federation of Gynecology and Obstetrics
NCCN: National Comprehensive Cancer Network
IHC: Immunohistochemistry
BMI: Body mass index
MSI-H: Microsatellite instability high
ESGO: Europe’s Leading Gynaecological Oncology Congress
MMRD: Mismatch repair deficient
