# Supplementary figures and images for: Comparison of clinical characteristics and prognosis between type I and type II endometrial cancer: a single-center retrospective study
Source: Discov Oncol. 2023 Nov 23;14:211. doi: 10.1007/s12672-023-00820-1 (PMC10667178; doi:10.1007/s12672-023-00820-1)

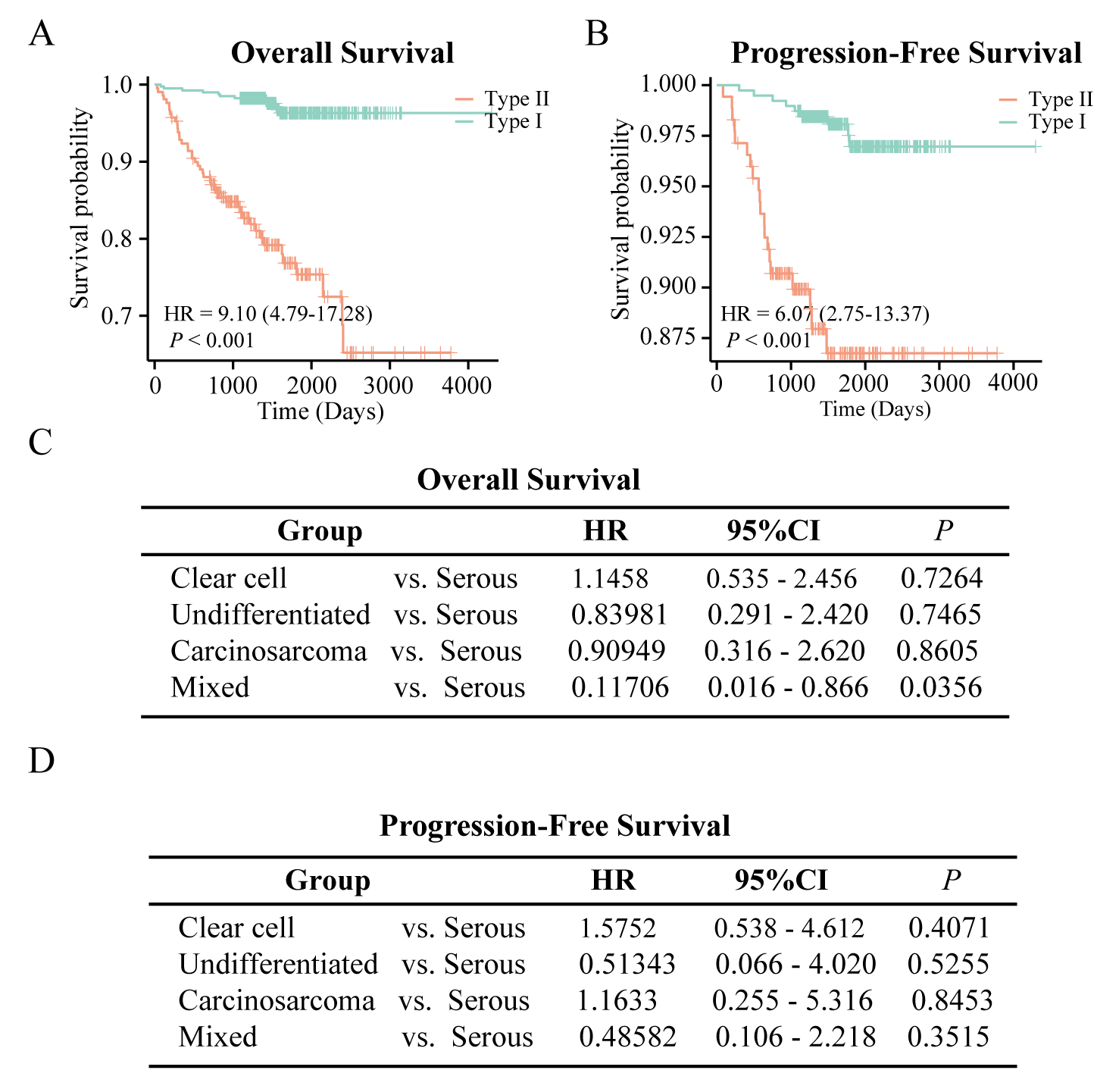

Supplement: Supplementary file 1 — Additional file1 (TIF 861 KB): Figure 1. Prognostic analysis of EC patients with different pathological type from our single center. (A) Prognostic difference between type I and type II EC patients regarding overall survival. (B) Prognostic difference between type I and type II EC patients regarding progression-free survival. (C) Prognostic difference between uterine serous carcinoma and other pathological types regarding overall survival. (D) Prognostic difference between uterine serous carcinoma and other pathological types regarding progression free survival [file 12672_2023_820_MOESM1_ESM.tif]
