# Supplementary material for: Comparison of clinical characteristics and prognosis between type I and type II endometrial cancer: a single-center retrospective study
Source: Discov Oncol. 2023 Nov 23;14:211. doi: 10.1007/s12672-023-00820-1 (PMC10667178; doi:10.1007/s12672-023-00820-1)
Supplement: Supplementary file 2 — Additional file2 (DOCX 23 KB) [file 12672_2023_820_MOESM2_ESM.docx]

**Supplementary Table 1. Univariate and multivariate Cox regression analysis for PFS in type I EC**

| **Characteristics** | **No.** | **Univariate analysis** | |  | **Multivariate analysis** | |
| --- | --- | --- | --- | --- | --- | --- |
|  |  | **Hazard ratio (95% CI)** | ***P-*value** |  | **Hazard ratio (95% CI)** | ***P-*value** |
| **Age** | 389 | 1.132 (1.060 - 1.210) | **< 0.001** |  | 1.154 (1.060 - 1.256) | **< 0.001** |
| **Menopause** | 389 |  |  |  |  |  |
| No | 148 | Reference |  |  |  |  |
| Yes | 230 | 4.989 (0.624 - 39.914) | 0.130 |  |  |  |
| Unknown | 11 | 0.000 (0.000 - Inf) | 0.998 |  |  |  |
| **BMI** | 123 | 0.698 (0.445 - 1.095) | 0.118 |  |  |  |
| **Chemotherapy alone** | 389 |  |  |  |  |  |
| No | 260 | Reference |  |  | Reference |  |
| Yes | 129 | 0.205 (0.025 - 1.651) | 0.136 |  | 0.119 (0.014 - 0.978) | **0.048** |
| **Chemoradiotherapy** | 389 |  |  |  |  |  |
| No | 356 | Reference |  |  |  |  |
| Yes | 33 | 3.113 (0.646 - 14.992) | 0.157 |  |  |  |
| **Without systemic therapy** | 389 |  |  |  |  |  |
| Yes | 224 | Reference |  |  |  |  |
| No | 165 | 0.561 (0.139 - 2.266) | 0.417 |  |  |  |
| **Stage** | 389 |  |  |  |  |  |
| I | 356 | Reference |  |  |  |  |
| II | 6 | 0.000 (0.000 - Inf) | 0.998 |  |  |  |
| III | 26 | 1.594 (0.199 - 12.767) | 0.660 |  |  |  |
| IV | 1 | 0.000 (0.000 - Inf) | 0.999 |  |  |  |
| **Myometrial infiltration (>1/2)** | 389 |  |  |  |  |  |
| No | 295 | Reference |  |  | Reference |  |
| Yes | 66 | 5.061 (1.354 - 18.921) | **0.016** |  | 0.129 (0.034 - 0.492) | **0.003** |
| Unknown | 28 | 0.000 (0.000 - Inf) | 0.999 |  | 0.000 (0.000 - Inf) | 0.997 |
| **Cervix involvement** | 389 |  |  |  |  |  |
| No | 328 | Reference |  |  | Reference |  |
| Yes | 18 | 8.931 (1.634 - 48.823) | **0.012** |  | 32.147 (6.163 - 167.688) | **< 0.001** |
| Unknown | 43 | 6.468 (1.438 - 29.094) | **0.015** |  | 151.027 (37.038 - 615.822) | **< 0.001** |
| **Lymph node metastasis** | 389 |  |  |  |  |  |
| No | 297 | Reference |  |  |  |  |
| Yes | 17 | 2.447 (0.301 - 19.898) | 0.403 |  |  |  |
| Unknown | 75 | 0.580 (0.071 - 4.715) | 0.610 |  |  |  |
| **Ascites cytology** | 389 |  |  |  |  |  |
| Negative | 369 | Reference |  |  | Reference |  |
| Positive | 8 | 0.000 (0.000 - Inf) | 0.998 |  | 0.000 (0.000 - Inf) | 0.998 |
| Unknown | 12 | 22.333 (3.729 - 133.744) | **< 0.001** |  | 13.390 (2.165 - 82.836) | **0.005** |

BMI: Body Mass Index; PFS: Progression Free Survival; CI: Confidence Interval.
