# Supplementary material for: Comparison of clinical characteristics and prognosis between type I and type II endometrial cancer: a single-center retrospective study
Source: Discov Oncol. 2023 Nov 23;14:211. doi: 10.1007/s12672-023-00820-1 (PMC10667178; doi:10.1007/s12672-023-00820-1)
Supplement: Supplementary file 3 — Additional file3 (DOCX 23 KB) [file 12672_2023_820_MOESM3_ESM.docx]

**Supplementary Table 2. Univariate and multivariate Cox regression analysis for PFS in type II EC.**

| **Characteristics** | **No.** | **Univariate analysis** | |  | **Multivariate analysis** | |
| --- | --- | --- | --- | --- | --- | --- |
|  |  | **Hazard ratio (95% CI)** | ***P-*value** |  | **Hazard ratio (95% CI)** | ***P*-value** |
| **Age** | 175 | 1.046 (0.992 - 1.102) | 0.096 |  | 1.041 (0.987 - 1.097) | 0.140 |
| **BMI** | 139 | 1.043 (0.917 - 1.187) | 0.519 |  |  |  |
| **Surgery** | 175 |  |  |  |  |  |
| No | 4 | Reference |  |  |  |  |
| Yes | 171 | 9220928.5243 (0.000 - Inf) | 0.997 |  |  |  |
| **Chemotherapy alone** | 175 |  |  |  |  |  |
| Yes | 64 | Reference |  |  |  |  |
| No | 111 | 1.357 (0.521 - 3.535) | 0.533 |  |  |  |
| **Radiotherapy alone** | 175 |  |  |  |  |  |
| No | 173 | Reference |  |  |  |  |
| Yes | 2 | 0.000 (0.000 - Inf) | 0.998 |  |  |  |
| **Chemoradiotherapy** | 175 |  |  |  |  |  |
| No | 103 | Reference |  |  |  |  |
| Yes | 72 | 1.780 (0.737 - 4.297) | 0.200 |  |  |  |
| **Stage** | 175 |  |  |  |  |  |
| I | 124 | Reference |  |  |  |  |
| II | 10 | 2.135 (0.473 - 9.640) | 0.324 |  |  |  |
| III | 31 | 2.389 (0.883 - 6.465) | 0.087 |  |  |  |
| IV | 6 | 2.123 (0.272 - 16.546) | 0.472 |  |  |  |
| Unknown | 4 | 0.000 (0.000 - Inf) | 0.997 |  |  |  |
| **Myometrial infiltration (>1/2)** | 175 |  |  |  |  |  |
| No | 107 | Reference |  |  | Reference |  |
| Yes | 56 | 3.820 (1.524 - 9.578) | **0.004** |  | 3.035 (1.130 - 8.150) | **0.028** |
| Unknown | 12 | 0.000 (0.000 - Inf) | 0.997 |  | 0.000 (0.000 - Inf) | 0.998 |
| **Cervix involvement** | 175 |  |  |  |  |  |
| No | 146 | Reference |  |  | Reference |  |
| Yes | 24 | 4.673 (1.908 - 11.444) | **< 0.001** |  | 2.424 (0.883 - 6.648) | 0.086 |
| Unknown | 5 | 0.000 (0.000 - Inf) | 0.998 |  | 0.306 (0.000 - Inf) | 1.000 |
| **Lymph node metastasis** | 175 |  |  |  |  |  |
| No | 128 | Reference |  |  | Reference |  |
| Yes | 29 | 3.228 (1.248 - 8.349) | **0.016** |  | 1.976 (0.673 - 5.807) | 0.215 |
| Unknown | 18 | 1.484 (0.329 - 6.703) | 0.608 |  | 1.640 (0.342 - 7.865) | 0.536 |
| **Ascites cytology** | 175 |  |  |  |  |  |
| Negative | 161 | Reference |  |  | Reference |  |
| Positive | 14 | 0.000 (0.000 - Inf) | 0.998 |  | 0.000 (0.000 - Inf) | 0.998 |

BMI: Body Mass Index; PFS: Progression Free Survival; CI: Confidence Interval.
