# Supplementary material for: Comparison of clinical characteristics and prognosis between type I and type II endometrial cancer: a single-center retrospective study
Source: Discov Oncol. 2023 Nov 23;14:211. doi: 10.1007/s12672-023-00820-1 (PMC10667178; doi:10.1007/s12672-023-00820-1)
Supplement: Supplementary file 4 — Additional file4 (DOCX 21 KB) [file 12672_2023_820_MOESM4_ESM.docx]

**Supplementary Table 3. Univariate and multivariate Cox regression analysis of type II EC in stage I regarding OS**

| **Characteristics** | **No.** | **Univariate analysis** | |  | **Multivariate analysis** | |
| --- | --- | --- | --- | --- | --- | --- |
|  |  | **Hazard ratio (95% CI)** | ***P-*value** |  | **Hazard ratio (95% CI)** | ***P-*value** |
| **Age** | 138 | 1.061 (1.004 - 1.121) | **0.037** |  | 1.089 (1.008 - 1.177) | **0.030** |
| **Menopause** | 138 |  |  |  |  |  |
| Yes | 124 | Reference |  |  |  |  |
| No | 13 | 0.433 (0.057 - 3.291) | 0.419 |  |  |  |
| Unknown | 1 | 0.000 (0.000 - Inf) | 0.998 |  |  |  |
| **BMI** | 109 | 1.320 (1.074 - 1.623) | **0.008** |  | 1.388 (1.083 - 1.780) | **0.010** |
| **Chemotherapy alone** | 138 |  |  |  |  |  |
| No | 84 | Reference |  |  |  |  |
| Yes | 54 | 0.512 (0.168 - 1.561) | 0.239 |  |  |  |
| **Chemoradiotherapy** | 138 |  |  |  |  |  |
| No | 90 | Reference |  |  |  |  |
| Yes | 48 | 0.588 (0.208 - 1.659) | 0.316 |  |  |  |
| **Myometrial infiltration (>1/2)** | 138 |  |  |  |  |  |
| No | 94 | Reference |  |  | Reference |  |
| Yes | 39 | 3.185 (1.255 - 8.081) | **0.015** |  | 2.648 (0.721 - 9.732) | 0.143 |
| Unknown | 5 | 0.000 (0.000 - Inf) | 0.997 |  | 0.000 (0.000 - Inf) | 0.999 |
| **Cervix involvement** | 138 |  |  |  |  |  |
| No | 135 | Reference |  |  |  |  |
| Yes | 2 | 0.000 (0.000 - Inf) | 0.998 |  |  |  |
| Unknown | 1 | 0.000 (0.000 - Inf) | 0.999 |  |  |  |
| **Ascites cytology** | 138 |  |  |  |  |  |
| No | 132 | Reference |  |  |  |  |
| Yes | 6 | 0.000 (0.000 - Inf) | 0.998 |  |  |  |

BMI: Body Mass Index; OS: Overall Survival; CI: Confidence Interval.
