# Supplementary material for: Comparison of clinical characteristics and prognosis between type I and type II endometrial cancer: a single-center retrospective study
Source: Discov Oncol. 2023 Nov 23;14:211. doi: 10.1007/s12672-023-00820-1 (PMC10667178; doi:10.1007/s12672-023-00820-1)
Supplement: Supplementary file 5 — Additional file5 (DOCX 21 KB) [file 12672_2023_820_MOESM5_ESM.docx]

**Supplementary Table 4. Univariate and multivariate Cox regression analysis of type II EC in stage I regarding PFS**

| **Characteristics** | **No.** | **Univariate analysis** | |  | **Multivariate analysis** | |
| --- | --- | --- | --- | --- | --- | --- |
|  |  | **Hazard ratio (95% CI)** | ***P-*value** |  | **Hazard ratio (95% CI)** | ***P-*value** |
| **Age** | 124 | 1.068 (0.993 - 1.148) | 0.075 |  | 1.076 (0.991 - 1.168) | 0.082 |
| **Menopause** | 124 |  |  |  |  |  |
| No | 12 | Reference |  |  |  |  |
| Yes | 112 | 1.040 (0.133 - 8.136) | 0.970 |  |  |  |
| Unknown | 0 |  |  |  |  |  |
| **BMI** | 101 | 1.145 (0.947 - 1.386) | 0.162 |  |  |  |
| **Chemotherapy alone** | 124 |  |  |  |  |  |
| No | 75 | Reference |  |  |  |  |
| Yes | 49 | 0.350 (0.075 - 1.624) | 0.180 |  |  |  |
| **Chemoradiotherapy** | 124 |  |  |  |  |  |
| Yes | 45 | Reference |  |  |  |  |
| No | 79 | 0.484 (0.147 - 1.590) | 0.232 |  |  |  |
| **Without systemic therapy** | 124 |  |  |  |  |  |
| No | 95 | Reference |  |  |  |  |
| Yes | 29 | 1.204 (0.319 - 4.544) | 0.784 |  |  |  |
| **Myometrial infiltration (>1/2)** | 124 |  |  |  |  |  |
| No | 88 | Reference |  |  | Reference |  |
| Yes | 31 | 3.669 (1.119 - 12.026) | **0.032** |  | 2.711 (0.717 - 10.257) | 0.142 |
| Unknown | 5 | 0.000 (0.000 - Inf) | 0.998 |  | 0.000 (0.000 - Inf) | 0.998 |
| **Cervix involvement** | 124 |  |  |  |  |  |
| No | 121 | Reference |  |  | Reference |  |
| Yes | 2 | 52.031 (9.408 - 287.767) | **< 0.001** |  | 23.937 (3.805 - 150.584) | **< 0.001** |
| Unknown | 1 | 0.000 (0.000 - Inf) | 0.999 |  | 1.045 (0.000 - Inf) | 1.000 |
| **Ascites cytology** | 124 |  |  |  |  |  |
| Negative | 118 | Reference |  |  |  |  |
| Positive | 6 | 0.000 (0.000 - Inf) | 0.998 |  |  |  |

BMI: Body Mass Index; PFS: Progression Free Survival; CI: Confidence Interval.
